# Weight misperception and substance use: Brazilian Study of Cardiovascular Risks in Adolescents (ERICA)

**DOI:** 10.1186/s12889-022-14267-6

**Published:** 2022-10-04

**Authors:** Simoni Urbano da Silva, Vivian Siqueira Santos Gonçalves, Laura Augusta Barufaldi, Kenia Mara Baiocchi de Carvalho

**Affiliations:** 1 Graduate Program of Public Health, Faculty of Health Sciences, University of Brasilia, Brasilia, DF Brazil; 2 Population Research Division, Brazilian National Cancer Institute José Alencar Gomes da Silva, Rio de Janeiro, RJ Brazil

**Keywords:** Body image, Weight misperception, Adolescent, Health surveys, Alcohol drinking, Cigarette smoking, Tobacco

## Abstract

**Background:**

Adolescence is a crucial period for body image formation. Weight misperception is the discrepancy between individuals’ body weight perception and their actual nutritional status. Both weight concerns and substance use are common among adolescents, and there is evidence of an associations between these two variables. Thus, the aim of this study was to assess the association between weight misperception and substance use (smoking and alcohol) in a national sample of normal weight Brazilian adolescents.

**Methods:**

Data were obtained from the Brazilian Study of Cardiovascular Risks in Adolescents (ERICA), a cross-sectional, multicenter, national, school-based survey, carried out in 124 municipalities with more than 100,000 inhabitants from Brazil. The sample included adolescents aged 12–17 years, classified as normal weight by nutritional status evaluation. The following measures were collected: weight underestimation and overestimation (exposure); having tried cigarette smoking, current smoking, current alcohol consumption, binge drinking and current smoking and alcohol consumption(outcomes); macro-region, sex, type of school, and excessive screen time (confounders). The frequency of variables was calculated with 95% confidence intervals (CI). Poisson regression models were used to estimate prevalence ratios (PR).

**Results:**

In total, data from 53,447 adolescents were analyzed. Weight misperception was present in a third of the adolescents, with similar prevalence of weight underestimation and overestimation. In adolescents aged 12–14 years, weight underestimation and overestimation were associated with having tried cigarette smoking (PR: 1.18 and 1.43, respectively), current alcohol consumption (PR: 1.33 for both weight misperception categories), and binge drinking (PR: 1.96 and 2.01, respectively). Weight underestimation was associated with both having tried cigarette smoking and current alcohol consumption in boys (PR: 1.14 and 1.16, respectively) and girls (PR: 1.32 and 1.15, respectively). In girls, weight overestimation was associated with all substance use variables (PR between 1.19 and 1.41).

**Conclusions:**

Our results showed an association between weight misperception and having tried cigarette smoking, alcohol consumption, and binge drinking in younger adolescents. In addition, weight overestimation was associated with all substance use indicators in girls. Based on our findings, interventions aimed to improve weight perception in normal weight adolescents may contribute to the reduction of substance use in this population.

**Supplementary Information:**

The online version contains supplementary material available at 10.1186/s12889-022-14267-6.

## Background

Adolescence is the period of life that encompasses the ages of 10–19 years. During this period, adolescents are undergoing rapid physical, cognitive, social, and emotional development [1]. It is a crucial phase for body image formation [2], as well as a stage during which individuals may be under pressure to consume substances such as cigarettes and alcohol [3].

Body image is a multidimensional construct, that involves an individual’s perception of their physical appearance (e.g, mental representations of their own body size) and their thoughts, feelings, and attitudes about their body [4]. Factors such as sex [4, 5], mass media [4, 6], and opinion of peers [4] have an important influence on body weight perception, particularly during childhood and adolescence.

Body image disorders include, among other aspects, a distorted self-perception [4]. Weight misperception exists when an individual perceives their body weight differently from their actual nutritional status [7, 8], and it can manifest as weight underestimation or overestimation, depending on the direction of the perceived inaccuracy regarding weight [9, 10]. Misclassification of one’s weight status has been associated with negative health consequences, such as unhealthy weight control behaviors [5], ultra-processed food consumption [9, 11], depressive symptoms [5] and also substance use [10, 12, 13].

Engaging in substance use, such as alcohol and tobacco, is considered a risk factor for chronic non-communicable diseases and social problems [14, 15]. The onset of alcohol drinking and smoking in adolescence is considered a public health concern, as it is believed that early initiation increases the chances of these habits persisting in adult life [16, 17]. During adolescence, alcohol consumption is related to school problems [18], violence [18, 19], risky sexual behaviors [19], use of other substances [20], and cognitive and neural damage [21]. Tobacco use, on the other hand, increases the risk of nicotine dependence [22] and has harmful effects on health [23]. Due to the harmful effects of alcohol and tobacco use in this age group, it is important to clarify the factors that predispose individuals to their consumption to elucidate how to prevent this behavior.

Both weight concerns and substance use are common occurrences among Brazilian adolescents. The prevalence of body weight underestimation and overestimation are 13.5–22.2% and 12.0–20.0%, respectively [24–26] across different study populations. Regarding substance use, the most recent evidence shows the following prevalence in Brazilian adolescents: 22.6% for having tried cigarette smoking, 6.8% for current smoking, 28.1% for current alcohol consumption, and 6.9% for binge drinking [27]. Substance use rates are generally higher in older adolescents [27, 28], public schools students [27], and residents of the South macro-region of Brazil [27–29].

Although the associations between body image concern and substance use have been described previously [30, 31], reports about the Brazilian adolescent population are still scarce [32–34], given the dynamics of the social environment and possible associations with new factors. Hence, the aim of this study was to assess the association between weight misperception and substance use (smoking and alcohol) in a national representative sample of normal weight Brazilian adolescents.

## Methods

The recommendations of Strengthening the Reporting of Observational Studies in Epidemiology (STROBE) statement were used to report this study [35].

### Study design

The data analyzed in this study were obtained from the Brazilian Study of Cardiovascular Risks in Adolescents (ERICA in Portuguese), a cross-sectional, multicenter, and national school-based survey that aimed to estimate the prevalence of cardiovascular risk factors in adolescents [36]. More information about the methodology of the ERICA, including the design, sample, participants, and all data collection, has been described in previous publications [36–38].

### Study size, setting and participants

The ERICA is a complex sample study, in which participants were selected from a population of adolescents aged 12–17 years from public and private schools located in urban and rural areas of Brazil’s state capitals and 246 cities with more than 100,000 inhabitants. The municipalities were divided into 32 geographic strata formed by the 26 Brazilian state capitals and the Federal District, and five groups with the eligible municipalities of each macro-region.

In each geographic stratum, schools were selected with a probability proportional to size (PPS). The ratio between the number of students and the distance from the state capital was used to calculate the size measure of each school This selection was performed considering the school area (urban or rural) and school governance (public or private). After this stage, three classes in each school were selected from combinations of class shifts (morning or afternoon) and years (the 7th, 8th, and 9th year of elementary school and the 1st, 2nd, and 3rd year of high school). In the classes selected, all students were invited to participate in the study.

Adolescents outside the 12–17-year age group, those with permanent or temporary deficiency, and pregnant adolescents were excluded from the study.

In total, 1251 schools from 124 municipalities were selected, including 102,327 eligible students. Details regarding participation loss and refusal to participate have been presented previously [38]. For the purposes of this study, only the adolescents who answered the self-answered questionnaire and who were evaluated as having normal weight in the assessment of nutritional status were included. This criterion was used to investigate the effect of weight misperception without the confounding effect of different weight categories. The accurate recognition of overweight or obesity status may help to motivate adolescents to adopt healthy habits [39], and thus weight underestimation in this group may have detrimental effects on health [40].

### Data source and variables

The ERICA data were collected from 2013 to 2014. Data on weight perception, smoking, alcohol consumption, screen time, and demographics were obtained from a self-administered questionnaire via a personal digital assistant (PDA), model LG GM750Q (LG Electronics, Seoul, Korea). Anthropometric measurements were collected using a portable stadiometer (Alturexata Inc., Minas Gerais, Brazil) to measure height, and a digital scale (Lider; São Paulo, Brazil) to measure weight. Appropriate and calibrated equipment were used.

The variables analyzed in this study were weight misperception and categories (exposure), substance use variables (outcomes), sociodemographic variables and screen time (confounders).

#### Weight misperception

Weight misperception was characterized by the disagreement between adolescents’ nutritional status and their self-perceived weight [8].

The body mass index (BMI)-for-age z-score cut-points of the World Health Organization were used for nutritional status definition, with different criteria by sex [41]. BMI was calculated as weight in kilograms divided by the square of height in meters.

Self-perceived weight was first measured by the question “Are you satisfied with your weight?” (yes or no). To the participants who answered “no” a second question followed: “In your opinion, at what level is your current weight?” (“below the ideal,” “above the ideal,” or “far above the ideal”). Adolescents with normal weight who classified themselves as “below the ideal” were assigned to the weight underestimation group. Those with a self-perception of “above the ideal” or “far above the ideal” were allocated to the weight overestimation group. Adolescents who presented weight underestimation or overestimation were considered to have weight misperception [9, 10].

#### Substance use

Having tried cigarette smoking was defined when the adolescent claimed to have tried smoking cigarettes in their lifetime. It was assessed using the question: “Have you ever tried smoking cigarettes, even one or two drags?” (Yes or No).

Current smoking was considered when the adolescents had smoked cigarettes in the past 30 days. This was measured with the question: “In the past 30 days, how many days have you smoked cigarettes?” (the answer options were dichotomized in “one or more days” or “No day/I never smoked cigarettes”).

Current alcohol consumption was defined when the adolescent had consumed alcoholic beverages in the past 30 days. It was measured with the question: “In the past 30 days, how many days have you taken at least one glass or a dose of alcoholic beverage?” (the answer options were dichotomized in “one or more days” or “No day/I never drank alcoholic beverages”).

Binge drinking in the past 30 days was defined when the adolescent consumed five or more drinks on the same occasion. It was measured with the question: “In the past 30 days, on the days you had an alcoholic drink, how many glasses or doses did you drink on average? (The answer options were dichotomized as “five glasses or doses or more in the past 30 days” or “I never drank alcoholic beverages/I didn’t drink alcoholic beverages in the past 30 days/Four glasses or doses or less in the past 30 days”).

Current smoking and alcohol consumption was a composited variable based on the questions used for variables that investigated current smoking and current alcohol consumption separately, to capture when the adolescent had both smoked cigarettes and consumed alcoholic beverages in the past 30 days.

#### Confounder variables

The demographic variables selected from the plausibility criterion were macro-region [27–29] (North, Northeast, Southeast, South or Midwest), sex [24, 40, 42] (boys or girls), age group [12, 42] (12–14 years or 15–17 years), and type of school [27] (public or private), which in this study was considered as an indicator of socioeconomic status due to the direct relationship between income and education expenses in Brazil [43].

Excessive screen time has been shown previously to be associated with smoking [44], alcohol consumption [45], and body image indicators [32, 46]. It was used in this study as a proxy for media exposure time [32, 44]. It was measured with the question: “On a common weekday, how many hours do you use a computer or watch television or play video games?” (according the relevant recommendations [47], the answer options were dichotomized as “2 or more hours per day “or “less than 2 hours per day/I don’t do this activity on a common weekday”).

### Bias

Several efforts were made in the ERICA study to ensure quality and minimize study bias. Prior to the data collection, a pilot study was conducted and the field researchers’ teams were carefully trained to standardize the procedures. During the data collection period, the database was automatically updated when information was entered into the PDA. Moreover, in order to detect outliers or discrepancies in the measurements, the data collection and systematization were monitored by local supervisors and by the coordination of the ERICA study through a central quality control system.

### Statistical methods

A theoretical model was developed from a literature review to define the analytical plan for this study. Software for Statistics and Data Science (Stata) [48], version 14.0 was used to perform the statistical analysis, using the “survey” command. As a complex sampling study, the natural weights and use of post-stratification estimators were considered in the analysis.

The frequency of variables was calculated with 95% confidence intervals (CI) according to the substance use indicators. In the association study, Poisson regression models were used to estimate prevalence ratios (PRs). A homogeneity analysis, using Mantel–Haenszel tests, was performed to test the interaction effect of sex and age group on the associations between weight misperception categories and substance.

In this analysis, the exposure variables were weight underestimation and weight overestimation. The outcomes were having tried cigarette smoking, current smoking, current alcohol consumption, current binge drinking and current smoking and alcohol consumption. The “no weight underestimation/overestimation” category was the reference group in all regression models.

The outcomes variables were tested separately for each exposure in a crude analysis. Only the prevalence ratios with a *p*-value < 0.20 were tested in the models adjusted for the confounder variables. Statistical significance was set at *p*-value < 0.05.

## Results

**Fig. 1:**
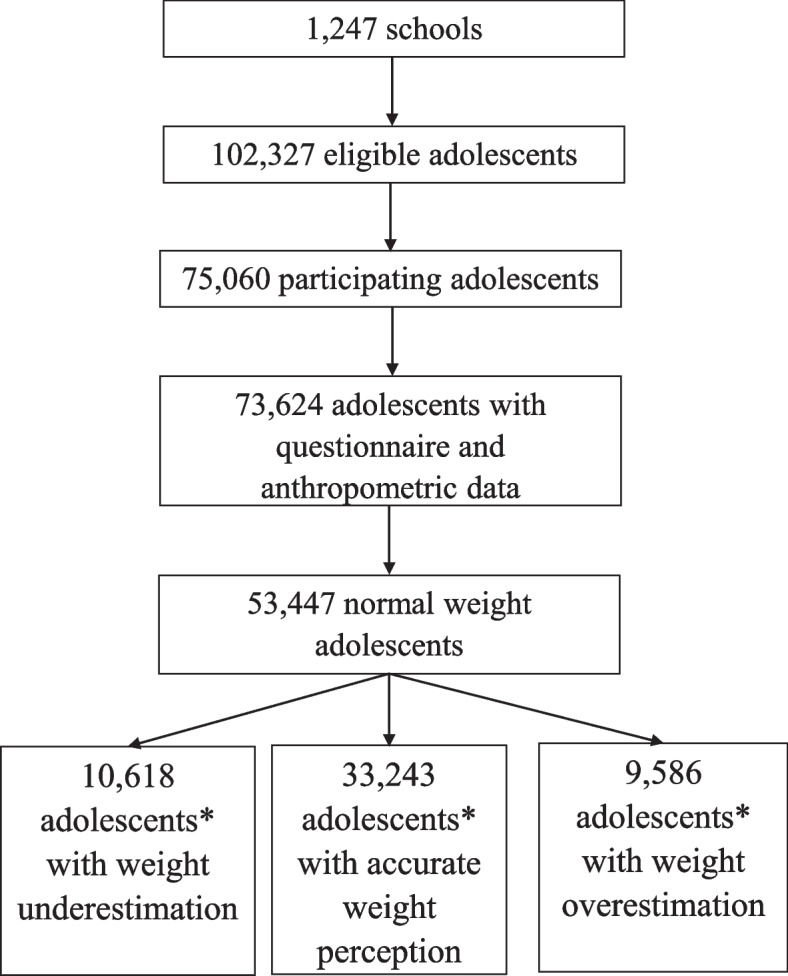
Enrollment of normal weight adolescents from the ERICA study, Brazil, 2013–2014. *Differences between the absolute numbers and percentages in results are explained by natural weights and post-stratification estimators used in statistical analysis

Of the ERICA sample, 75,060 students from 1247 schools were investigated in some stage of the data collection, and 73,624 answered the self-administered questionnaire and had their anthropometric measurements collected. A total of 53,447 adolescents had normal weight according to the nutritional status definition and were analyzed in this study (Fig. 1).

Table 1 presents the participants’ characteristics according to the substance use condition. Weight misperception was present in a third of the adolescents, with similar rates of weight underestimation and weight overestimation. Adolescents in the 15–17 year age group presented a higher prevalence of all substance use studied. Current alcohol consumption and binge drinking were more prevalent in adolescents with excessive screen time (24.0 and 4.6%, respectively).

**Table 1:** Descriptive statistics of the normal weight participants from ERICA study, Brazil, 2013–2014

Characteristics	Outcomes										Total	
	Having tried cigarette smoking		Current smoking		Current alcohol consumption		Current binge drinking		Current smoking and alcohol			
	%	(95% CI)	%	(95% CI)	%	(95% CI)	%	(95% CI)	%	(95% CI)	%	(95% CI)
**Age group**												
12–14 years	11.0	10.0–12.1	3.2	2.7–3.8	14.1	13.1–15.1	1.5	1.2–1.9	2.2	1.8–2.8	52.7	^a^
15–17 years	**26.8**	**24.9–28.7**	**6.6**	**5.9–7.4**	**29.0**	**27.0–31.0**	**6.3**	**5.6–7.1**	**5.0**	**4.3–5.7**	**47.3**	^a^
**Sex**												
Male	18.9	17.7–20.2	5.3	4.7–5.9	21.1	19.5–22.8	4.1	3.6–4.6	3.7	3.2–4.3	50.2	^a^
Female	18.0	16.6–19.4	4.4	3.8–5.0	21.2	19.8–22.6	3.5	3.0–4.1	3.3	2.8–3.9	49.8	^a^
**Race/ethnicity**												
White	17.2	15.6–18.9	4.5	3.8–5.2	22.1	20.4–23.9	4.0	3.4–4.7	3.5	3.0–4.2	39.1	37.5–40.8
Black or brown	19.3	18.1–20.5	5.0	4.4–5.7	20.8	19.5–22.1	3.7	3.2–4.2	3.6	3.0–4.3	**58.0**	**56.3–59.6**
Indigenous or Asian	16.2	13.4–19.4	5.0	3.4–7.3	17.6	14.7–20.8	2.4	1.7–3.5	3.2	2.1–4.8	2.9	2.6–3.3
**Macro-region**												
North	19.3	18.3–20.4	4.9	4.3–5.6	14.9	13.7–16.1	3.3	2.8–3.8	3.2	2.7–3.7	8.4	^a^
Northeast	15.5	14.2–16.9	4.0	3.3–4.8	16.3	14.7–18.1	3.7	3.2–4.2	2.5	2.0–3.1	21.3	^a^
Southeast	18.2	16.4–20.1	4.7	4.0–5.5	22.2	20.4–24.2	3.6	3.0–4.4	3.5	2.9–4.3	50.8	^a^
South	22.6	19.7–25.7	6.6	5.0–8.7	**28.3**	**25.3–31.5**	4.6	3.7–5.7	5.5	3.9–7.7	11.8	^a^
Midwest	20.9	19.4–22.4	4.9	4.1–5.9	23.0	21.3–24.8	4.4	3.8–5.2	3.8	3.1–4.6	7.7	^a^
**Type of school**												
Public	**19.2**	**18.0–20.4**	4.9	4.5–5.4	20.9	19.7–22.1	3.5	3.1–4.0	3.6	3.1–4.0	**83.8**	**79.4–87.4**
Private	14.5	11.8–17.8	4.1	2.8–6.0	22.4	19.1–26.2	**5.1**	**4.3–6.1**	3.4	2.3–5.2	16.2	12.6–20.6
**Screen time > 2 hours/day**												
No	17.5	16.1–19.1	4.5	3.9–5.3	18.1	16.6–19.7	2.9	2.5–3.4	3.3	2.7–4.0	48.6	47.2–50.1
Yes	19.3	17.9–20.8	5.1	4.4–5.8	**24.0**	**22.9–25.2**	**4.6**	**4.1–5.2**	3.8	3.2–4.4	51.4	49.9–52.8
**Weight misperception**												
No	16.6	15.5–17.7	4.3	3.9–4.8	19.1	18.0–20.2	3.3	2.9–3.7	3.1	2.7–3.5	**66.0**	**65.0–66.9**
Yes	**22.1**	**20.7–23.6**	**5.7**	**5.0–6.6**	**25.1**	**23.6–26.8**	**4.8**	**4.1–5.4**	**4.4**	**3.7–5.2**	34.0	33.1–35.0
**Weight underestimation**												
No	17.5	16.5–18.5	4.7	4.2–5.2	20.4	19.3–21.5	3.6	3.2–4.0	3.4	2.9–3.9	**82.9**	**82.3–83.5**
Yes	**23.1**	**21.3–25.0**	5.5	4.7–6.4	**24.7**	**22.7–26.8**	**4.9**	**4.1–5.9**	4.1	3.4–5.0	17.1	16.5–17.7
**Weight overestimation**												
No	17.9	16.8–19.0	4.6	4.1–5.1	20.2	19.2–21.3	3.6	3.3–4.0	3.3	2.9–3.7	**83.0**	**82.0–84.0**
Yes	**21.1**	**19.4–22.9**	5.9	4.8–7.4	**25.6**	**23.6–27.7**	4.6	3.8–5.5	4.7	3.5–6.1	16.9	16.0–18.0

Significant difference between categories frequency were shown in bold. ^a^ Variables used to calculate the natural weights and calibration factors of the sample

Adolescents with weight misperception presented a higher prevalence of having tried cigarette smoking (22.1%), current smoking (5.7%), current alcohol consumption (25.1%), current binge drinking (4.8%) and current smoking and alcohol (4.4%). Those adolescents who underestimated their weight presented a higher prevalence of having tried cigarette smoking (23.1%), current consumption of alcohol (24.7%) and current binge drinking (4.9%). About a fifth of adolescents with weight overestimation had smoked cigarette recently, and a quarter of them currently consumed alcohol (Table 1).

There was an interaction effect between age group and sex on the associations between weight misperception and most of the substance use indicators studied. Thus, the Poisson regression models were stratified according to these variables.

According to the adjusted analysis results, the presence of weight underestimation in adolescents with 12–14 years was directly associated with having tried cigarette smoking, current alcohol consumption, current binge drinking, and current smoking and alcohol, with PR varying from 1.18 to 1.96. Additionally, the presence of weight overestimation was also associated with having tried cigarette smoking (PR: 1.44), current alcohol consumption (PR: 1.33) and current binge drinking (PR: 2.01) in this age group. In older adolescents (15–17 years), weight underestimation was associated with having tried cigarette smoking (PR: 1.24), and weight overestimation was associated with current alcohol consumption (PR: 1.15) (Table 2).

**Table 2:** Weight misperception and substance use, by age group, in adolescents from ERICA study, Brazil, 2013–2014

		12–14 years				15–17 years			
		Crude		Adjusted ^a^		Crude		Adjusted ^a^	
		PR ^b^(95% CI)	***P***-value	PR ^b^ (95% CI)	*P*-value	PR ^b^(95% CI)	*P*-value	PR^**b**^ (95% CI)	*P*-value
**Weight underestimation**									
	Having tried cigarette smoking	1.19 (1.04–1.36)	0.013	**1.18 (1.03–1.35)**	0.020	1.22 (1.13–1.32)	< 0.001	**1.24 (1.14–1.35)**	< 0.001
**Outcomes**	Current smoking	1.18 (0.86–1.62)	0.313	*	*	1.08 (0.89–1.31)	0.452	*	*
	Current alcohol consumption	1.32 (1.14–1.53)	< 0.001	**1.33 (1.14–1.56)**	< 0.001	1.05 (0.96–1.15)	0.271	*	*
	Current binge drinking	1.99 (1.18–3.35)	0.009	**1.96 (1.14–3.36)**	0.015	1.08 (0.88–1.32)	0.456	*	*
	Current smoking and alcohol^3^	1.44 (1.00–2.07)	0.050	**1.49 (1.05–2.11)**	0.027	1.05 (0.83–1.33)	0.666	*	*
**Weight overestimation**									
	Having tried cigarette smoking	1.39 (1.13–1.71)	0.002	**1.43 (1.14–1.80)**	0.002	1.04 (0.92–1.18)	0.499	*	*
**Outcomes**	Current smoking	1.60 (0.94–2.73)	0.086	1.74 (0.95–3.19)	0.075	1.11 (0.89–1.39)	0.359	*	*
	Current alcohol consumption	1.37 (1.18–1.59)	< 0.001	**1.33 (1.12–1.58)**	0.001	1.17 (1.05–1.30)	0.004	**1.15 (1.04–1.27)**	0.008
	Current binge drinking	2.08 (1.27–3.38)	0.003	**2.01 (1.30–3.10)**	0.002	1.01 (0.82–1.25)	0.899	*	*
	Current smoking and alcohol^c^	1.78 (0.87–3.64)	0.113	1.88 (0.79–4.48)	0.153	1.30 (0.98–1.72)	0.067	1.30 (0.97–1.76)	0.083

Significant differences in adjusted prevalence ratios were shown in bold. ^a^Adjusted by macro-region, sex, type of school and screen time. ^b^ Prevalence ratio. ^c^This sample was composed only by adolescents who had current smoking and alcohol consumption and those who had not consumed both these substances in the last 30 days. *The *P*-value in crude analysis was ≥0.20

In analyses stratified by sex, weight underestimation was directly associated with both having tried cigarette smoking and current alcohol consumption in boys (PR: 1.14 and 1.16, respectively) and girls (PR: 1.32 and 1.15, respectively). No associations between weight overestimation and substance use indicators were found in boys. Nonetheless, weight overestimation was associated with having tried cigarette smoking, current smoking, current alcohol consumption, current binge drinking, and current smoking and alcohol in girls, with PR varying from 1.19 to 1.41 (Table 3).

**Table 3:** Weight misperception and substance use, by sex, in adolescents from ERICA study, Brazil, 2013–2014

		Male				Female			
		Crude		Adjusted^**a**^		Crude		Adjusted^**a**^	
		PR^**b**^(95% CI)	*P*-value	PR^**b**^ (95% CI)	*P*-value	PR^**b**^(95% CI)	*P*-value	PR^**b**^ (95% CI)	*P*-value
**Weight underestimation**									
	Having tried cigarette smoking	1.26 (1.13–1.41)	< 0.001	**1.14 (1.02–1.27)**	0.020	1.38 (1.25–1.53)	< 0.001	**1.32 (1.20–1.45)**	< 0.001
**Outcomes**	Current smoking	1.27 (1.00–1.61)	0.046	1.17 (0.92–1.49)	0.187	1.08 (0.85–1.37)	0.521	*	*
	Current alcohol consumption	1.24 (1.07–1.45)	0.005	**1.16 (1.00–1.35)**	0.048	1.17 (1.04–1.32)	0.007	**1.15 (1.03–1.29)**	0.013
	Current binge drinking	1.50 (1.14–1.96)	0.003	1.24 (0.92–1.66)	0.150	1.25 (0.96–1.63)	0.093	1.17 (0.89–1.53)	0.251
	Current smoking and alcohol^3^	1.39 (1.04–1.85)	0.026	1.26 (0.94–1.68)	0.119	1.11 (0.82–1.49)	0.499	*	*
**Weight overestimation**									
	Having tried cigarette smoking	1.12 (0.89–1.42)	0.320	*	*	1.25 (1.13–1.39)	< 0.001	**1.19 (1.06–1.35)**	0.004
**Outcomes**	Current smoking	1.39 (0.71–2.72)	0.340	*	*	1.42 (1.09–1.83)	0.008	**1.35 (1.04–1.74)**	0.024
	Current alcohol consumption	1.20 (1.01–1.43)	0.036	1.16 (0.97–1.39)	0.094	1.32 (1.19–1.47)	< 0.001	**1.22 (1.10–1.36)**	< 0.001
	Current binge drinking	0.93 (1.62–1.39)	0.713	*	*	1.58 (1.22–2.04)	0.001	**1.41 (1.07–1.85)**	0.013
	Current smoking and alcohol^c^	1.64 (0.74–3.61)	0.223	*	*	1.54 (1.14–2.08)	0.005	**1.39 (1.04–1.85)**	0.027

Significant differences in adjusted prevalence ratios were shown in bold. ^a^Adjusted by macro-region, sex, type of school and screen time. ^b^ Prevalence ratio. ^c^This sample was composed only by adolescents who had current smoking and alcohol consumption and those who had not consumed both these substances in the last 30 days. *The *P*-value in crude analysis was ≥0.20

## Discussion

To our knowledge, this is the first study to investigate the association between weight misperception and substance use in adolescents in a representative sample from the largest Brazilian cities. Considering the relevance of possible associations between weight misperception and substance use at a critical stage of behavior development, our results showed a direct association between both weight misperception categories and the substance use variables examined, especially in younger adolescents and among girls. Our findings are consistent with prior results found in South Korea [13], the United States of America [10, 49], and China [12], corroborating the hypothesis that there is a direct association between weight misperception and cigarette and alcohol use in adolescents.

The high prevalence of substance use found in this study in older adolescents was expected and consistent with the results of the previous Brazilian national surveillance data collected in 2015 and 2019 [27, 42]. Similarly, body image concerns, such as weight misperception and body dissatisfaction, are also more frequent in older adolescents [50, 51]. However, the greater magnitude of the associations found between weight misperception and the substance use in younger adolescents deserves attention, particularly the higher prevalence of binge drinking in both weight misperception categories in this age group. It must be considered that although adolescence is a well-characterized period, the predictors of smoking and drinking can be different in early, middle, and late adolescence [52, 53].

A possible explanation for the association with tobacco smoking indicators is the belief that smoking can help control body weight. According to previous studies, concerns regarding body weight [30], beliefs about weight control effects [54, 55], and perceived attractiveness [56, 57] are related to smoking, more so in adolescents with self-perception of being overweight [55, 58]. Even so, adolescents who self-perceived themselves as underweight may see smoking habits as a mechanism to maintain a weight status [44].

Alcohol consumption is related to self-esteem in youth [59, 60]. Adolescents who misperceived their weight demonstrated a higher prevalence of current alcohol consumption and binge drinking, possibly attempting to increase their self-esteem. In a previous study, Winter and collaborators [56] hypothesized that adolescent boys might use alcohol and tobacco to sustain their friendships and social status. However, the relationship between body weight and alcohol consumption is complex: the presence of caloric restriction or purging combined with a binge drinking episode has been described as a way to avoid weight gain from excess calories, and this phenomenon has been named in the non-medical terms, as “drunkorexia” and “alcoholimia” [61]. Alternatively, alcohol consumption may be a tactic for coping with emotional distress resulting from binge eating episodes [62].

Additionally, the differences by sex in our results are of interest. As observed in our study, girls who perceive themselves as overweight are more likely to smoke and consume alcohol compared to boys [49, 56, 58]. This finding could be related to beliefs about the weight management effects of cigarettes [54, 55] and influence of alcohol on self-esteem [59, 60], as mentioned above. Usually, girls are more likely to overestimate their weight, whereas boys are more likely to underestimate it [5, 12, 63]. According to a previous Brazilian study with similar sample [51], the prevalence of weight overestimation was higher in girls, however there was no difference in weight underestimation prevalence between sexes. Regarding substance use, the prevalence of current smoking [27, 28, 64] and alcohol consumption [29] in Brazilian adolescents are similar for both sexes according to most recent surveys.

The association between weight misperception categories and the joint consumption of cigarettes and alcohol in younger adolescents and girls reinforces the importance of considering factors associated with polysubstance use in adolescence. The consumption of alcohol or cigarettes separately is harmful on the adolescents’ health [20, 22]. However, the concurrent consumption of tobacco and alcohol in adolescence is related a depression symptoms [65] and drugs use in adulthood [66].

In our results, adolescents who reported excessive screen time, used as a proxy for media exposure, demonstrated higher prevalence of alcohol consumption. Still, this phenomenon was not observed for cigarette smoking. It is known that alcohol marketing influences both alcohol consumption and binge drinking in young people [67, 68]. Perhaps, the differences in screen time exposure can be explained by stricter regulation of tobacco product marketing in Brazil [69], compared to alcoholic beverage advertising regulations [70]. As a consequence of smoking control policies [69], there was decreases in the prevalence of smoking over the last few years in Brazil, both in the adult [71] and adolescent [64] populations. However, an increase in binge drinking has been observed in adults between 2006 and 2019 [72], although among adolescents there was a slight decrease of current alcohol consumption between 2009 and 2015 [64].

Apart from the association between weight misperception and substance use demonstrated in this study, weight misperception in adolescents has other negative health consequences. In previous studies, weight overestimation was associated with unhealthy weight control strategies, such as use of diet medications, purging, or fasting, and disordered eating behaviors in children and adolescents [73, 74]. On the other hand, weight underestimation was associated with inappropriate weight gain behaviors in boys [13]. Overall, there is evidence that adolescents with weight misperception are more likely to gain excessive weight over time [75, 76], which highlights the need to promote accurate weight perception in this age group.

Given the relevance of the results of this investigation, it is important to consider the interventions that have been effective in improving body image in adolescents. According to a systematic review of school-based universal body image programs, interventions in younger participants and using media literacy were most effective. Interventions including activities to improve self-esteem and discussed peer influence on body image were also effective [77]. In Brazil, a validated protocol called *Body Project* reduced the thin-ideal internalization and increased body appreciation in girls with weight concerns and criteria for eating disorders [78]. A study protocol that aims to improve body image in Brazilian adolescents was recently published. The intervention, called *Topity,* is based on digital intervention through a social media chatbot using therapeutic techniques [79]. Their results had not been published at the time of writing this manuscript.

Our study has several strengths. The national and large sample of ERICA, as well as the methodological care in the sample design, data collection, and analysis reinforce the reliability of the present study. Another point worth mentioning is the measurement of anthropometric data by trained researchers using validated methods. Furthermore, the use of a PDA in data collection was important for adolescents’ privacy, particularly for questions that were sensitive in nature, such as substance use.

However, this study also has limitations. First, participants of our sample were adolescents with normal weight who attended school in cities with more than 100,000 inhabitants. Therefore, the results cannot be generalized to the entire Brazilian adolescent population. The limitations of self-answered questionnaires should also be mentioned. Although this type of questionnaire avoids the interviewer’s interference, the participant may not understand the questions and/or the answer options. Third, although the questions used in the assessment the weight misperception indicators were similar to those used in other surveys, the ERICA study was not a specific to body image; thus, a multidimensional approach to measure this variable was not used. Fourth, other possible interfering factors, such as physical activity, mental health, food consumption, and eating disorders, were not considered in this analysis. Fifth, the ERICA study did not investigate the screen time on weekends, which may limit the use of the indicator as a proxy for cigarette and alcoholic beverages media exposure time. Finally, the cross-sectional design of the study makes it impossible to establish a cause-and-effect relationship between weight misperception and substance use.

## Conclusions

Our results corroborate the hypothesis that there is an association between weight misperception and substance use in Brazilian adolescents. A direct association was found between both categories of weight misperception and most of the substance use variables in younger adolescents. Weight underestimation was associated with both having tried cigarette smoking and current alcohol consumption in boys and girls. In addition, in adolescent girls, there was an association between weight overestimation and all substance use studied.

Based on our results, the implementation of programs aimed at reinforcing a positive body image among adolescents is recommended. Improvements in accurate perceptions in normal weight adolescents may contribute to the reduction of substance use in this population. Furthermore, investment in longitudinal research is also recommended to establish the trajectories of weight misperception, smoking initiation, and onset of alcohol consumption. Thus, universal interventions aimed at promoting a positive body image and preventing substance use in adolescents can be enhanced.

## Supplementary Information

**Additional file 1.** Dataset used in this study.

**Additional file 2.** Dictionary of dataset variables used in this study.

## Data Availability

All data generated or analyzed during this study are included in this published article [and its supplementary information files].

## Abbreviations

BMI: body mass index
CI: confidence interval
ERICA: Brazilian Study of Cardiovascular Risks in Adolescents
PDA: personal digital assistant
PPS: probability proportional to size
PR: prevalence ratio
Stata: Software for Statistics and Data Science
STROBE: Strengthening the Reporting of Observational Studies in Epidemiology

## References

[1] World Health Organization. Global Accelerated Action for the Health of Adolescents (AA-HA!): Guidance to support country implementation. 2017. https://www.who.int/publications/i/item/9789241512343. Accessed 15 Dec 2021.

[2] Voelker DK, Reel JJ, Greenleaf C (2015). Weight status and body image perceptions in adolescents: current perspectives. Adolesc Health Med Ther.

[3] Jones A, Winter VR, Pekarek E, Walters J (2018). Binge drinking and cigarette smoking among teens: does body image play a role?. Child Youth Serv Rev.

[4] Cash TF, Cash TF (2012). Cognitive-behavioral perspectives on body image. Encyclopedia of Body Image and Human Appearance.

[5] Lee J, Lee Y (2016). The association of body image distortion with weight control behaviors, diet behaviors, physical activity, sadness, and suicidal ideation among Korean high school students: A cross-sectional study. BMC Public Health.

[6] Holland G, Tiggemann M (2016). A systematic review of the impact of the use of social networking sites on body image and disordered eating outcomes. Body Image.

[7] Schlissel AC, Schwartz TT, Skeer MR (2017). The association between body image and behavioral misperception (BIBM) and alcohol use among high school girls: results from the 2013 youth risk behavioral survey. J Stud Alcohol Drugs.

[8] Duncan DT, Wolin KY, Scharoun-Lee M, Ding EL, Warner ET, Bennett GG (2011). Does perception equal reality? Weight misperception in relation to weight-related attitudes and behaviors among overweight and obese US adults. Int J Behav Nutr Phys Act.

[9] Lim H, Wang Y (2013). Body weight misperception patterns and their association with health-related factors among adolescents in South Korea. Obesity (Silver Spring).

[10] Jiang Y, Kempner M, Loucks EB (2014). Weight misperception and health risk behaviors in youth: the 2011 US YRBS. Am J Health Behav.

[11] Angoorani P, Heshmat R, Ejtahed HS (2017). Body weight misperception and health-related factors among Iranian children and adolescents: the CASPIAN-V study. J Pediatr Endocrinol Metab.

[12] Xie B, Chou CP, Spruijt-Metz D (2006). Weight perception and weight-related sociocultural and behavioral factors in Chinese adolescents. Prev Med (Baltim).

[13] Shin A, Nam CM (2015). Weight perception and its association with socio-demographic and health-related factors among Korean adolescents. BMC Public Health.

[14] World Health Organization. Alcohol. 2018. http://www.who.int/news-room/fact-sheets/detail/alcohol. Acessed 23 Jan 2022.

[15] World Health Organization. Tobacco. 2021. https://www.who.int/news-room/fact-sheets/detail/tobacco. Acessed 23 Jan 2022.

[16] Haardörfer R, Windle M, Fairman RT, Berg CJ (2021). Longitudinal changes in alcohol use and binge-drinking among young-adult college students: analyses of predictors across system levels. Addict Behav.

[17] Dutra LM, Glantz SA (2018). Thirty-day smoking in adolescence is a strong predictor of smoking in young adulthood. Prev Med.

[18] Conegundes LSO, Valente JY, Martins CB, Andreoni S, Sanchez ZM (2020). Binge drinking and frequent or heavy drinking among adolescents: prevalence and associated factors. J Pediatr.

[19] Gomes K, Amato T de C, Bedendo A, Dos Santos EL, Noto AR. Problems associated with binge drinking among students in Brazil’s state capitals. Cienc e Saude Coletiva 2019;24(2):497–507. 10.1590/1413-81232018242.35452016. 10.1590/1413-81232018242.35452016 30726382

[20] Halladay J, Woock R, El-Khechen H, Munn C, MacKillop J, Amlung A (2020). Patterns of substance use among adolescents: A systematic review. Drug Alcohol Depend.

[21] Lees B, Meredith LR, Kirkland AE, Bryant BE, Squeglia LM (2020). Effect of alcohol use on the adolescent brain and behavior. Pharmacol Biochem Behav.

[22] Ebrahimi Kalan M, Bahelah R, Bursac Z, Ben Taleb Z, DiFranza JR, Tleis M (2020). Predictors of nicotine dependence among adolescent waterpipe and cigarette smokers: A 6-year longitudinal analysis. Drug Alcohol Depend.

[23] Onor ICO, Stirling DL, Williams SR (2017). Clinical effects of cigarette smoking: epidemiologic impact and review of pharmacotherapy options. Int J Environ Res Public Health.

[24] Andrade VMB, de Santana MLP, Fukutani KF, Queiroz ATL, Arriaga MB, Damascena NF (2020). Systems nutrology of adolescents with divergence between measured and perceived weight uncovers a distinctive profile defined by inverse relationships of food consumption. Nutrients..

[25] Araújo CL, Dumith SC, Menezes AMB, Hallal PC (2010). Peso medido, peso percebido e fatores associados em adolescentes. Rev Panam Salud Pública.

[26] Moehlecke M, Blume CA, Cureau FV, Kieling C, Schaan BD (2020). Self-perceived body image, dissatisfaction with body weight and nutritional status of Brazilian adolescents: A nationwide study. J Pediatr.

[27] Instituto Brasileiro de Geografia e Estatística. Pesquisa Nacional de Saúde Do Escolar (PeNSE) 2019. 2021. https://biblioteca.ibge.gov.br/index.php/biblioteca-catalogo?view=detalhes&id=2101852. Acessed 20 Jan 2022.

[28] Figueiredo VC, Szklo AS, Costa LC, Kuschnir MCC, Silva TLN, Bloch KV (2016). ERICA: smoking prevalence in Brazilian adolescents. Rev Saude Publica.

[29] Coutinho ESF, França-Santos D, Silva Magliano E, Bloch KV, Barufaldi LA, Cunha CF (2016). ERICA: patterns of alcohol consumption in Brazilian adolescents. Rev Saude Publica.

[30] Potter BK, Pederson LL, Chan SSH, Aubut JAL, Koval JJ (2004). Does a relationship exist between body weight, concerns about weight, and smoking among adolescents? An integration of the literature with an emphasis on gender. Nicotine Tob Res.

[31] Field AE, Austin SB, Frazier AL, Gillman MW, Camargo CA, Colditz GA (2002). Smoking, getting drunk, and engaging in bulimic behaviors: in which order are the behaviors adopted?. J Am Acad Child Adolesc Psychiatry.

[32] Miranda VPN, Amorim PRS, Bastos RR, Souza VGB, Faria ER, Franceschini SCC (2021). Body image disorders associated with lifestyle and body composition of female adolescents. Public Health Nutr.

[33] Guimarães BE de B, Aquino R, Prado NM de BL, Rodrigues PVA. O consumo excessivo de álcool e a insatisfação com a imagem corporal por adolescentes e jovens de um município baiano, Brasil. Cad Saude Publica. 2019;36(1):e00044919. 10.1590/0102-311X044919. 10.1590/0102-311X044919 31939544

[34] Duarte L, Fujimori E, Borges AL, Kurihayashi A, Steen M, Lay AR (2020). Body weight dissatisfaction is associated with cardiovascular health-risk behaviors among brazilian adolescents: findings from a national survey. Int J Environ Res Public Health.

[35] Von EE, Altman DG, Egger M, Pocock SJ, Gøtzsche PC, Vandenbroucke JP (2008). The strengthening the reporting of observational studies in epidemiology (STROBE) statement : guidelines for reporting observational studies. J Clin Epidemiol.

[36] Bloch KV, Szklo M, Kuschnir MC, Abreu GA, Barufaldi LA, Klein CH (2015). The study of cardiovascular risk in adolescents - ERICA: rationale, design and sample characteristics of a national survey examining cardiovascular risk factor profile in Brazilian adolescents. BMC Public Health.

[37] Vasconcellos MTL, Silva PLN, Szklo M, Kuschnir MCC, Klein CH, Abreu GA, et al. Sampling design for the Study of Cardiovascular Risk in Adolescents (ERICA). 2015;31(5):1–10. 10.1590/0102-311X00043214. 10.1590/0102-311X00043214 26083168

[38] Silva TLN, Klein CH, Moura Souza A, Barufaldi LA, Abreu GA, Kuschnir MCC, et al. Response rate in the study of cardiovascular risks in adolescents - ERICA. Rev Saude Publica. 2016;50 Supl 1. 10.1590/S01518-8787.2016050006730. 10.1590/S01518-8787.2016050006730 PMC4767034 26910552

[39] Pasch KE, Klein EG, Laska MN, Velazquez CE, Moe SG, Lytle LA (2011). Weight misperception and health risk behaviors among early adolescents. Am J Health Behav.

[40] San Martini MC, De Assumpção D, Barros MBDA, Barros Filho ADA, Mattei J (2021). Weight self-perception in adolescents: evidence from a population-based study. Public Health Nutr.

[41] World Health Organization. Growth Reference 5–19 Years 2007. https://www.who.int/tools/growth-reference-data-for-5to19-years. Acessed 25 June 2021.

[42] Malta DC, Machado IE, Felisbino-Mendes MS, Prado RR, Pinto AMS, Oliveira-Campos M (2018). Uso de substâncias psicoativas em adolescentes brasileiros e fatores associados: Pesquisa Nacional de Saúde dos Escolares, 2015. Rev Bras Epidemiol..

[43] Instituto Brasileiro de Geografia e Estatística. Pesquisa de Orçamentos Familiares 2017–2018: Primeiros resultados. 2019. https://biblioteca.ibge.gov.br/visualizacao/livros/liv101670.pdf. Acessed 15 Aug 2021.

[44] Leatherdale ST, Wong SL, Manske SR, Colditz GA (2008). Susceptibility to smoking and its association with physical activity, BMI, and weight concerns among youth. Nicotine Tob Res.

[45] West AB, Bittel KM, Russell MA, Evans MB, Mama SK, Conroy DE (2020). A systematic review of physical activity, sedentary behavior, and substance use in adolescents and emerging adults. Transl Behav Med.

[46] Tebar WR, Canhin DS, Colognesi LA, Morano AEVA, Silva DTC, Christofaro DGD. Body dissatisfaction and its association with domains of physical activity and of sedentary behavior in a sample of 15,632 adolescents. Int J Adolesc Med Health. 2020. 10.1515/ijamh-2019-0241. 10.1515/ijamh-2019-0241 32549167

[47] Expert panel on integrated guidelines for cardiovascular health and risk reduction in children and adolescents; National Heart, Lung, and Blood Institute. Expert panel on integrated guidelines for cardiovascular health and risk reduction in children and adolescents: summary report. Pediatrics. 2011;128(Suppl 5):S213–56. 10.1542/peds.2009-2107C. 10.1542/peds.2009-2107C PMC4536582 22084329

[48] StataCorp. Stata Statistical Software: Release 14. 2015.

[49] Akomolafe TO, Hansen AR, Hackney AA, Wang W, Thorne-Williams DR, Zhang J. Weight misperception and cigarette smoking among healthy weight adolescents in the U. S: NHANES 2005–2014. J Child Adolesc Subst Abus 2020;0(0):1–8. 10.1080/1067828X.2020.1774025.

[50] Tebar WR, Gil FCS, Scarabottolo CC, Codogno JS, Fernandes RA, Christofaro DGD (2020). Body size dissatisfaction associated with dietary pattern, overweight, and physical activity in adolescents: A cross-sectional study. Nurs Health Sci.

[51] Silva SU, Alves MA, Vasconcelos FAG, Gonçalves VSS, Barufaldi LA, Carvalho KMB (2021). Association between body weight misperception and dietary patterns in Brazilian adolescents: cross-sectional study using ERICA data. PLoS One.

[52] O’Loughlin J, O’Loughlin EK, Wellman RJ (2017). Predictors of cigarette smoking initiation in early, middle, and late adolescence. J Adolesc Health.

[53] Burk WJ, van der Vorst H, Kerr M, Stattin H (2012). Alcohol use and friendship dynamics: selection and socialization in early-, middle-, and late-adolescent peer networks. J Stud Alcohol Drugs..

[54] Pénzes M, Czeglédi E, Balázs P, Foley KL (2012). Factors associated with tobacco smoking and the belief about weight control effect of smoking among Hungarian adolescents. Cent Eur J Public Health.

[55] Cawley J, Dragone D, Scholder SVHK (2014). The demand for cigarettes as derived from the demand for weight loss: A theoretical and empirical investigation.

[56] Ramseyer Winter V, Kennedy AK, O’Neill E. Adolescent Tobacco and Alcohol Use: The Influence of Body Image. J Child Adolesc Subst Abus. 017;26(3):219–228. 10.1080/1067828X.2017.1279992

[57] Kilibarda B, Gudelj Rakic J, Mitov Scekic S, Krstev S (2020). Smoking as a weight control strategy of Serbian adolescents. Int J Public Health.

[58] Yoon J, Bernell SL (2016). Link between perceived body weight and smoking behavior among adolescents. Nicotine Tob Res.

[59] Wu CST, Wong HT, Shek CHM, Loke AY (2014). Multi-dimensional self-esteem and substance use among Chinese adolescents. Subst Abus Treat Prev Policy.

[60] Lee CG, Seo DC, Torabi MR, Lohrmann DK, Song TM (2018). Longitudinal trajectory of the relationship between self-esteem and substance use from adolescence to young adulthood. J Sch Health.

[61] Thompson-Memmer C, Glassman T, Diehr A (2019). Drunkorexia: A new term and diagnostic criteria. J Am Coll Heal.

[62] Nieri T, Kulis S, Keith VM, Hurdle D (2005). Body image, acculturation, and substance abuse among boys and girls in the southwest. Am J Drug Alcohol Abuse.

[63] Jankauskiene R, Baceviciene M (2019). Body image concerns and body weight overestimation do not promote healthy behaviour: evidence from adolescents in Lithuania. Int J Environ Res Public Health.

[64] Oliveira-Campos M, Oliveira MM, Silva SU, Santos MAS, Barufaldi LA, Oliveira PPV (2018). Risk and protection factors for chronic noncommunicable diseases in adolescents in Brazilian capitals. Rev Bras Epidemiol.

[65] Williams GC, Patte KA, Ferro MA, Leatherdale ST (2021). Associations between longitudinal patterns of substance use and anxiety and depression symptoms among a sample of Canadian secondary school students. Int J Environ Res Public Health.

[66] Moss HB, Chen CM, Yi H, ye. (2014). Early adolescent patterns of alcohol, cigarettes, and marijuana polysubstance use and young adult substance use outcomes in a nationally representative sample. Drug Alcohol Depend.

[67] Jernigan D, Noel J, Landon J, Thornton N, Lobstein T (2017). Alcohol marketing and youth alcohol consumption: A systematic review of longitudinal studies published since 2008. Addiction..

[68] Finan LJ, Lipperman-Kreda S, Grube JW, Balassone A, Kaner E. Alcohol marketing and adolescent and young adult alcohol use behaviors: A systematic review of cross-sectional studies. J Stud Alcohol Drugs Suppl. 2020; Suppl 19:42–56. 10.15288/jsads.2020.s19.42. 10.15288/jsads.2020.s19.42 PMC7063997 32079561

[69] Portes LH, Machado CV, Turci SRB, Figueiredo VC, Cavalcante TM, Costa e Silva VL. (2018). Tobacco control policies in Brazil: A 30-year assessment. Cienc e Saude Coletiva..

[70] Oliveira CWL, Mendes CV, Kiepper A, Monteiro MG, Wagner GA, Sanchez ZM (2021). Analysis of gaps in alcohol policies in Brazil using the Pan American health Organization’s alcohol policy scoring. Int J Drug Policy.

[71] Malta DC, Silva AGD, Machado ÍE, Sá ACMGN, Santos FMD, Prates EJS (2019). Trends in smoking prevalence in all Brazilian capitals. J Bras Pneumol.

[72] Malta DC, Silva AG da, Prates EJS, Alves FTA, Cristo EB, Machado ÍE. Convergência no consumo abusivo de álcool nas capitais brasileiras entre sexos, 2006 a 2019: O que dizem os inquéritos populacionais. Rev Bras Epidemiol. 2021;24 Suppl 1. 10.1590/1980-549720210022.supl.1.

[73] Haynes A, Kersbergen I, Sutin A, Daly M, Robinson E (2018). A systematic review of the relationship between weight status perceptions and weight loss attempts, strategies, behaviours and outcomes. Obes Rev.

[74] Lim H, Lee HJ, Park S, Kim CI, Joh HK, Oh SW (2014). Weight misperception and its association with dieting methods and eating behaviors in south Korean adolescents. Nutr Res Pract.

[75] Cuypers K, Kvaløy K, Bratberg G, Midthjell K, Holmen J, Holmen TL. Being normal weight but feeling overweight in adolescence may affect weight development into young adulthood - an 11-year followup: the HUNT study, Norway. J Obes. 2012;2012. 10.1155/2012/601872. 10.1155/2012/601872 PMC3362140 22666556

[76] Sutin AR, Terracciano A (2015). Body weight misperception in adolescence and incident obesity in young adulthood. Psychol Sci.

[77] Yager Z, Diedrichs PC, Ricciardelli LA, Halliwell E (2013). What works in secondary schools? A systematic review of classroom-based body image programs. Body Image..

[78] Amaral ACS, Stice E, Ferreira MEC. A controlled trial of a dissonance-based eating disorders prevention program with Brazilian girls. Psicol Reflex e Crit. 2019;32(1). 10.1186/s41155-019-0126-3. 10.1186/s41155-019-0126-3 PMC6967323 32026167

[79] Matheson EL, Smith HG, Amaral ACS, Meireles JFF, Almeida MC, Mora G (2021). Improving body image at scale among Brazilian adolescents: study protocol for the co-creation and randomised trial evaluation of a chatbot intervention. BMC Public Health.

