# Supplementary material for: Weight misperception and substance use: Brazilian Study of Cardiovascular Risks in Adolescents (ERICA)
Source: BMC Public Health. 2022 Oct 4;22:1850. doi: 10.1186/s12889-022-14267-6 (PMC9531377; doi:10.1186/s12889-022-14267-6)
Supplement: Supplementary file 2 — Additional file 2. Dictionary of dataset variables used in this study. [file 12889_2022_14267_MOESM2_ESM.pdf]

Weight misperception and substance use: A cross-sectional study using data from the Brazilian Study of Cardiovascular Risks in Adolescents (ERICA)

Short title: Weight misperception and substances in adolescents

Simoni Urbano da Silva[1], Vivian Siqueira Santos Gonçalves[1], Laura Augusta Barufaldi[2], Kenia Mara Baiocchi de Carvalho[1,3]

[1] Graduate Program of Public Health, Faculty of Health Sciences, University of Brasilia, Brasilia, DF, Brazil.

[2] Population Research Division, Brazilian National Cancer Institute José Alencar Gomes da Silva, Rio de Janeiro, RJ, Brazil.

[3] Graduate Program in Human Nutrition, Faculty of Health Sciences, University of Brasilia, Brasilia, DF, Brazil.

Dictionary of dataset variables used

**Variable**	**Label**	**Code**
cod_UPA	Primary sampling unit code	Sample weighting variables
cod_stra_sel	Sample selection stratum code	Sample weighting variables
natweight	Natural weight of the design that represents the probability of inclusion in the sample	Sample weighting variables
poststrat	Post-stratum	Post-stratification variables
postweight	Post-weight	Post-stratification variables
id_erica	Participant identification number	Continuous variable
weightsatisfaction	Student's satisfaction with their own weight	1 = Yes<br>2 = No
weightperception	Opinion about current weight	1 = Below the ideal<br>2 = Above the ideal<br>3 = Far above the ideal

weightkg	Weight in kilograms	Continuous variable
height1	1st height measurement in centimeters	Continuous variable
height2	2st height measurement in centimeters	Continuous variable
heightaverage	Average between the two height measurements	Continuous variable
bmi	Body Mass Index in kg/m²	Continuous variable
zescore_bmi	Z-score of BMI-for-age	Continuous variable
nutritionalstatus	Nutritional status classification, according to BMI-for-age z-scores (WHO, 2007)	3 = Normal weight*<br><br>*In this study, only adolescents with normal weight were analyzed
weightmisperception	Presence of weight misperception	0 = No<br>1 = Yes
underestimation	Presence of weight underestimation	0 = No<br>1 = Yes
overestimation	Presence of weight overestimation	0 = No<br>1 = Yes
sex	Sex	0 = Female<br>1 = Male
macroregion	Brazilian macro-region	0 = North<br>1 = Northeast<br>2 = Southeast<br>3 = South<br>4 = Midwest
typeschool	Type of school	0 = Public<br>1 = Private
agegroup	Age group	0 = 12 -14 years old<br>1 = 15 -17 years old

ethnicity	Race/ethnicity	0 = White<br>1 = Black or brown<br>2 = Indigenous or Asian
screentime	Time spent using the computer, watching television or playing video games > 2 hours/day	0 = No<br>1 = Yes
expcigarette	To have tried smoking cigarettes in lifetime	0 = No<br>1 = Yes
currentsmoking	To have smoked cigarettes in the past 30 days	0 = No<br>1 = Yes
currentalcohol	To have consumed alcoholic beverages in the past 30 days	0 = No<br>1 = Yes
bingedrink	To have consumed five or more drinks on the same occasion in the past 30 days	0 = No<br>1 = Yes
smokingalcohol	To have both smoked cigarettes and consumed alcoholic beverages in the past 30 days	0 = No<br>1 = Yes
